# Association Between Adherence to 24-Hour Movement Guidelines and Noncommunicable Disease Risk in Chinese Adults: Prospective Cohort Study

**DOI:** 10.2196/47517

**Published:** 2024-03-27

**Authors:** Siyi Huang, Yuxuan Gu, Shahmir H Ali, Jingjing Xue, Ronghua Zhang, Xu Wen

**Affiliations:** 1 Department of Sports Science College of Education Zhejiang University Hangzhou China; 2 Nanjing Normal University Nanjing China; 3 Department of Social and Behavioral Sciences School of Global Public Health New York University New York, NY United States; 4 School of Humanities Beijing Dance Academy Beijing China; 5 Department of Nutrition and Food Safety Zhejiang Provincial Center for Disease Control and Prevention Hangzhou China

**Keywords:** chronic diseases, 24-hour movement guidelines, obesity, noncommunicable disease, sleep, risk, overweight

## Abstract

**Background:**

The increasing annual global deaths are attributable to noncommunicable chronic diseases (NCDs). Adhering to healthy lifestyle behaviors is associated with lower NCD risk, particularly among individuals with ample movement, enough sleep, and reduced sedentariness. Nevertheless, there are only few prospective assessments on the association of interactions between daily activities with NCD prevention, while the associations between adhering to Canadian 24-Hour Movement Guidelines (24HGs) for adults and NCD risks are still unknown. Compared to the general population, obese and overweight populations are at a higher risk of developing NCDs. Currently, it is unclear whether the health benefits of adhering to 24HGs differ between the general population and the obese population.

**Objective:**

This study explores prospective associations between adherence to 24HGs and NCD risks by weight status among overweight and obese adults in China.

**Methods:**

This decadal study consists of 9227 adults aged 35 years and older without any major NCDs at enrolment in the China Health and Nutrition Survey (2004-2011) and followed up until 2015. The exposure of interest was the overall score of compliance with 24HGs measured by participants’ self-report, wherein 1 point was assigned for compliance to each component, resulting in an aggregated score ranging from 0 to 3. The primary outcome was the first occurrence of major NCDs (high blood pressure, stroke, diabetes, cancer, and acute myocardial infarction). Log-binomial regression models were used to evaluate the associations.

**Results:**

<strong>:</strong> Overall, 4315 males and 4912 females, with 25,175 person-years of follow-up, were included in our analyses. The average baseline age was 50.21 (SD 11.04) years. Among the overweight and obese groups, those adhering to 1 (risk ratio [RR] 0.37, 95% CI 0.19-0.74; *P*=.004), 2 (RR 0.37, 95% CI 0.19-0.72; *P*=.003), and 3 (RR 0.32, 95% CI 0.14-0.73; *P*=.006) recommendations of 24HGs had a significantly lower NCD risk than those not adhering to any of the activity guidelines. Among the normal or underweight groups, those adhering to 1 (RR 0.49, 95% CI 0.27-0.96; *P*=.03) and 3 (RR 0.40, 95% CI 0.17-0.94; *P*=.03) components had a significantly lower NCD risk than those not adhering to any of the activity guidelines.

**Conclusions:**

In this prospective study, we found that active adherence to recommendations of 24HGs was associated with lower risks of NCDs, especially among overweight and obese participants. Additionally, overweight and obese individuals who met at least 1 component of 24HGs were at a significantly lower risk for NCDs, but this protective effect was not found among individuals in the normal and underweight groups. Individuals with excess body weight who tend to be more susceptible to health risks may gain greater health benefits than the general population by adhering to the recommendations of 24HGs.

## Introduction

In 2021, 71% of deaths worldwide (41 million) were attributable to noncommunicable chronic diseases (NCDs) [1]. Premature deaths due to NCDs were due to cardiovascular disease (17.9 million), cancer (9.3 million), respiratory diseases (4.1 million), and diabetes (1.5 million). Individuals with NCDs have a shorter life expectancy, struggle with disability, and experience a lower quality of life [2]. Several studies have shown that adhering to healthy lifestyle behaviors contributes to a reduced NCD risk. For example, 2 recent longitudinal studies [2,3] revealed that adhering to healthy lifestyle behaviors at midlife is associated with longer life expectancy and living free from NCDs, particularly if individuals practice at least 3 healthy lifestyle behaviors (ie, ample movement, enough sleep, reduced sedentariness). Among modifiable lifestyle factors, physical activity, limited leisure screen time, and adequate sleep duration have been consistently shown to have a protective effect against NCDs [4-7]. A meta-analysis evaluating the association between the levels of physical activity and the incidence of breast cancer showed that the relative risk of developing breast cancer was only 0.88 among women with the highest level of daily physical activity compared to those with the lowest level of daily physical activity [4]. Regarding sleep, either short or long sleep duration have been correlated with an increased risk of cancer, cardiovascular disease, and diabetes [5-7]. The available evidence from the UK Biobank has suggested that daily screen viewing, particularly television viewing and computer use, is associated with a modest, but significant, increase in the risk of esophagogastric and colon cancers [8]. Although the association between NCD risk and physical activity, sleep traits, or leisure screen time has been well-documented, only few longitudinal investigations have assessed the onset of NCDs in relation to long-term patterns of daily movements.

The global population has more than 19 billion overweight and 650 million obese individuals as of 2016 [9]. Overweight and obese individuals are at a higher risk of NCDs, losing at least 3.9 years of life expectancy compared with the general population [10]. Additionally, obesity is linked to immunologic dysfunction, which is detrimental to both innate and adaptive immune responses [11,12]. As such, strategies to reduce overweight and obesity could have a positive impact on the health of billions of individuals at risk of NCDs.

There are daily movement patterns associated with obesity and NCDs. For instance, exercise may help prevent NCDs by controlling blood pressure, preventing impaired glucose tolerance, and improving C-reactive protein levels [13]. Although some studies have investigated the association between the time spent on daily movement and risks of NCDs in overweight and obese populations, it remains unclear as to what extent adhering to recommendations on daily movement facilitates long-term benefits in preventing NCDs. Moreover, it is currently unclear whether the health benefits of adhering to recommended behaviors differ between the general population and obese populations.

To address these research gaps, this study prospectively assesses the association between the reports of overweight and obese patients on their behavioral patterns and their risk of NCDs by using the data of Chinese adults enrolled in the China Health and Nutrition Survey (CHNS). As a metric of behavioral patterns, we used the Canadian 24-Hour Movement Guidelines (24HGs) for adults, which measures participant compliance to physical activity, screen time, and sleep duration.

## Methods

### Study Population

Data were collected from CHNS, which is an ongoing, longitudinal, nationwide survey initiated in 1989 among a sample of Chinese residents aged ≥2 years from 9 provinces, with follow-ups every 2-4 years. Structured questionnaires were used to obtain data, including demographic, lifestyle, and health factors, from study participants by trained staff. A physical examination was conducted to collect anthropometric data. Details on the full questionnaire and methodology of data collection have been described elsewhere [14]. The survey methodology and data collection procedures were approved by the institutional review board of the University of North Carolina and the National Institute for Nutrition and Health, Chinese Center for Disease Control and Prevention (2015017). A longitudinal cohort analysis of the 2004-2015 CHNS data with complete publicly available daily movement and health data was chosen because it was after 2004 that the CHNS was used to collect sedentary time data in front of the screen. All NCD-free adults (age≥35 years) with complete data on height, weight, physical activity, screen time, chronic disease data, and sleep duration in a survey year were considered eligible participants. Only 1 baseline timepoint was used for the exposure variables to maximize the sample sizes and time intervals between exposure and potential outcomes. The final analyses included 9227 eligible participants.

### Measurement of Adherence to 24HGs

The exposure of interest was the overall scores of compliance with the Canadian 24HGs for adults (age 18-64 years) and older adults (65+ years), which were measured at baseline for all participants. Compliance was measured through adherence to 3 components of 24HGs: (1) adequate moderate to vigorous physical activity (MVPA) (≥150 min/week), (2) limited screen time (≤3 hours/day), and (3) recommended sleep duration (7-9 hours for adults aged 18-64 years and 7-8 hours for those older than 65 years). Using these baseline 24HGs data, we calculated an aggregated adherence score for each participant, ranging from 0-3 depending on how many of the aforementioned 24HGs components were met. Participants’ average daily time on MVPA was calculated using the reported time they spent on physical activities, commuting, and housework per day. We used the Compendium of Energy Expenditures for Adults to describe the absolute intensity of physical activities and estimate the metabolic equivalents for different kinds of exercises. The compendium defines activities involving energy expenditure >3 metabolic equivalents as MVPA. Participants in this study were classified as meeting physical activity guidelines when they accumulated an average of at least 150 minutes of MVPA per week, and 1 point was assigned to them. For sleep duration, participants reported the whole time spent on bed, covering daytime and nighttime. The total hours of all-day sleeping were recorded on average per day. According to 24HGs, uninterrupted sleep of 7-9 hours (age 18-64 years) and 7-8 hours per night (age ≥65 years) is considered as meeting the sleep duration recommendation, and 1 point was assigned to them. To measure leisure screen time, participants reported the average time spent on different recreational screen activities on a typical weekend and on a weekday. Participants were classified within the “recommended screen time” group if they spent less than 3 hours/day both on weekends and weekdays.

### Measurement of Weights and BMI

Weight and height were measured by trained staff following standardized protocols and performed at the same location as well as by following the same protocol at each survey visit. Weight was measured to the nearest 0.1 kg by using a calibrated beam scale while wearing lightweight clothing. Height was measured to the nearest 0.1 cm without wearing shoes by using a portable stadiometer. BMI was calculated as weight (kg) divided by the square of height (meter). The Working Group on Obesity in China proposed a threshold value for Chinese adults to judge the degree of overweight and obesity in 2003. This overweight and obesity metric is based on large-scale survey data of the Chinese population, wherein overweight was defined as BMI≥24 kg/m^2^ and obesity was defined as BMI≥28 kg/m^2^ [15].

### Assessment of Covariates

Demographic characteristics consisted of age, sex (male/female), household register (rural/urban), region (northern, southern, western, eastern, and, central China), baseline year (2004/2006, 2009/2011), energy intake (kcal), regular intake of sugar-sweetened beverage (more than 3 times per week or not), regular intake of tea (more than 3 times per week or not), regular intake of coffee (more than 3 times per week or not), smoking status (yes/ever/never), drinking alcohol status (yes/ever/never), light physical activities, family net income (in yuan; ¥1= US $0.15), and highest education level (graduation from lower middle school degree or lower/upper middle school degree, technical or vocational degree/university or college degree or higher). According to the level of economic development and regional characteristics, we divided the regions into 5 groups: eastern region (Shanghai, Jiangsu, and Shandong), western region (Chongqing and Guizhou), southern region (Guangxi), northern region (Beijing, Liaoning, and Heilongjiang), and central region (Henan, Hubei, and Hunan). Family income was inflated to 2015 and adjusted for regional differences.

### Ascertainment of NCDs

The primary outcome was the first occurrence of major NCDs, including high blood pressure, stroke, diabetes, cancer, and acute myocardial infarction. In each wave, participants were asked, “Have you ever been diagnosed with high blood pressure/stroke/diabetes/cancer/acute myocardial infarction?” Thus, in each survey wave, participants self-reported their health status, drawing from their recollection of hospital diagnoses and medical records. We obtained information pertaining to the presence of chronic diseases among individuals in the current wave. We considered participants to have NCDs as long as they had at least one of these NCDs.

### Ethics Approval

Written informed consent was obtained from all the study participants, and the CHNS received institutional review board approval from the University of North Carolina (No.2015017). The CHNS data sets are openly accessible, and the data are freely available to scholars in the CHNS official websites after submitting applications. Our research was based on approved deidentified data, and this study was approved by the medical ethics committee of the Department of Psychological and Behavioral Sciences at Zhejiang University (approval 2022060).

### Patient and Public Involvement Statement

Patients or the public were not involved in the design, conduct, reporting, or dissemination plans of our investigations. The CHNS data sets and published papers are available to the public online [16].

### Statistical Methods

Baseline characteristics were described using frequencies (percentages) and mean (SD). The characteristic differences of the study population were compared across meeting 24HGs by using chi-square tests for categorical variables and analysis of variance for continuous variables. Given the potential subjectivity in outcome collection, Cox proportional-hazards models were not deemed suitable for our investigation. At the same time, since overdispersion happens in Poisson regression, log-binomial regression models were used, which offer better convergence and directly provides us with the risk ratio (RR). RRs and 95% CIs for NCDs associated with 24HGs were estimated with the use of log-binomial regression models.

We evaluated the associations of adhering to recommendations with the incidence of NCDs by using log-binomial regression models with stepwise adjustment of confounding variables. Multivariate models were adjusted for age, age*age, and sex in model 1. Additionally, these were adjusted for the type of household register, province, survey year, smoking status, alcohol status, frequency of drinking sugar-sweetened beverages/tea/coffee, family net income, and light physical activity in model 2, and adjusted for the whole movement time in model 3. We imputed missing data by using multiple imputations [17] for continuous variables and the mode for the missing data of categorical variables. Adding the square of the variable allows us to model more accurately the effect of age, which may have a nonlinear relationship with our independent variable [18]. We applied component analysis—the ratio of the time spent to the recommended time for each activity based on the Physical Activity CoDa Regression Model and code [19].

In the sensitivity analysis considering the influence of specific recommendations, we assessed the association between adhering to a specific combination of 24HGs and the onset of chronic diseases among overweight/obese participants. In addition, we repeated the analysis after excluding underweight individuals at baseline. We also conducted subgroup analyses by using negative binomial regression analysis to explore whether the corresponding associations varied by age (35-49 years/≥50 years), sex (male/female), education level (low/high), and family income (low/high). We also tested the moderation effect of adhering to guidelines and the group variables. Analyses were performed with the R statistical package (version 4.2.2) [20]. All *P* values were 2-sided, with statistical significance set at <.05.

## Results

### Baseline Characteristics of the Study Population

Overall, 9227 participants (4315 males and 4912 females), with 25,175 person-years of follow-up, were included in our analyses, and the average baseline age was 50.21 (SD 11.04) years, with the majority of the participants being normal or underweight (5459/9227, 59.2%), living in rural environments (6124/9227, 66.4%), from Central or Eastern regions (4854/9227, 52.6%), and surveyed in 2004 and 2006 (6159/9227, 66.7%). As shown in Table 1, in terms of compliance with the guidelines, 112 (1.2%) participants did not meet any of the recommendations at baseline, and 2171 (23.5%), 6716 (72.8%), and 228 (2.5%) participants met 1, 2, and all components of the S4HGs, respectively. Compared to participants who met none, those who adhered to the overall recommendations of the guidelines were more likely to be older, live in urban environments, have lower energy intake, drink less coffee, never smoke, never drink alcohol, have a higher family net income, and have higher education.

**Table 1:** Characteristics of the participants by the category of adhering to the 24-hour movement guidelines at baseline.

Characteristic		Overall (N=9227)	Category of adhering to 24-hour movement guidelines					
			Meeting none (n=112)	Meet 1 standard (n=2171)	Meet 2 standards (n=6716)	Meet all standards (n=228)	*P* value	
Age at baseline (years), mean (SD)		50.21 (11.04)	49.01 (10.15)	53.57 (12.51)	49.11 (10.35)	51.38 (9.79)	<.001	
**Sex, n (%)**								.054
	Male	4315 (46.8)	62 (55.4)	1016 (46.8)	3146 (46.8)	91 (39.9)		
	Female	4912 (53.2)	50 (44.6)	1155 (53.2)	3570 (53.2)	137 (60.1)		
**BMI category, n (%)**								.01
	Normal weight	5072 (55)	62 (55.4)	1196 (55.1)	3695 (55)	119 (52.2)		
	Overweight	3093 (33.5)	30 (26.8)	697 (32.1)	2278 (33.9)	88 (38.6)		
	Obesity	675 (7.3)	15 (13.4)	164 (7.6)	479 (7.1)	17 (7.5)		
	Underweight	387 (4.2)	5 (4.5)	114 (5.3)	264 (3.9)	4 (1.8)		
**Household register, n (%)**								<.001
	Urban	3103 (33.6)	58 (51.8)	772 (35.6)	2129 (31.7)	144 (63.2)		
	Rural	6124 (66.4)	54 (48.2)	1399 (64.4)	4587 (68.3)	84 (36.8)		
**Region^a^, n (%)**								<.001
	Northern	1950 (21.1)	29 (25.9)	407 (18.7)	1445 (21.5)	69 (30.3)		
	Eastern	2273 (24.6)	24 (21.4)	529 (24.4)	1647 (24.5)	73 (32)		
	Central	2581 (28)	39 (34.8)	665 (30.6)	1837 (27.4)	40 (17.5)		
	Southern	979 (10.6)	2 (1.8)	192 (8.8)	767 (11.4)	18 (7.9)		
	Western	1444 (15.6	18 (16.1)	378 (17.4)	1020 (15.2)	28 (12.3)		
**Baseline year, n (%)**								<.001
	2004 and 2006	6159 (66.7)	68 (60.7)	1419 (65.4)	4561 (67.9)	111 (48.7)		
	2009 and 2011	3068 (33.3)	44 (39.3)	752 (34.6)	2155 (32.1)	117 (51.3)		
Total energy intake (kcal/day), mean (SD)		2136.22 (703.25)	2091.14 (657.13)	2079.97 (663.95)	2160.60 (717.64)	1973.80 (603.33)	<.001	
**Regular intake of sugar-sweetened beverage^b^, n (%)**								.31
	No	6953 (75.4)	90 (80.4)	1636 (75.4)	5068 (75.5)	159 (69.7)		
	Yes	2251 (24.4)	22 (19.6)	531 (24.5)	1629 (24.3)	69 (30.3)		
	Missing	23 (0.2)	0 (0)	4 (0.2)	19 (0.3)	0 (0)		
**Regular intake of tea^b^, n (%)**								<.001
	No	9006 (97.6)	104 (92.9)	2100 (96.7)	6586 (98.1)	216 (94.7)		
	Yes	202 (2.2)	8 (7.1)	65 (3)	118 (1.8)	11 (4.8)		
	Missing	19 (0.2)	0 (0)	6 (0.3)	12 (0.2)	1 (0.4)		
**Regular intake of coffee^b^, n (%)**								<.001
	No	3650 (96.8)	42 (93.3)	819 (95.1)	2688 (97.5)	101 (96.2)		
	Yes	112 (3)	3 (6.7)	41 (4.8)	64 (2.3)	4 (3.8)		
	Missing	6 (0.2)	0 (0)	1 (0.1)	5 (0.2)	0 (0)		
**Smoking status, n (%)**								<.001
	Never	6159 (66.7)	61 (54.5)	1404 (64.7)	4520 (67.3)	174 (76.3)		
	Yes/ever	3056 (33.1)	51 (45.5)	765 (35.2)	2186 (32.5)	54 (23.7)		
	Missing	12 (0.1)	0 (0)	2 (0.1)	10 (0.1)	0 (0)		
**Alcohol status, n (%)**								.04
	Never	5988 (64.9)	56 (50)	1409 (64.9)	4381 (65.2)	142 (62.3)		
	Yes/ever	3230 (35)	56 (50)	761 (35.1)	2327 (34.6)	86 (37.7)		
	Missing	9 (0.1)	0 (0)	1 (0)	8 (0.1)	0 (0)		
Light physical activities (h/wk), mean (SD)		14.02 (15.45)	11.70 (13.32)	14.44 (15.58)	13.91 (15.46)	14.93 (14.96)	.34	
Family net income (yuan/year; ¥1=US $0.15), mean (SD)		28,091.31 (36,727.18)	37,634.68 (42,909.64)	27,428.48 (37,254.09)	27,412.03 (35,500.69)	49,847.82 (53,384.15)	<.001	
**Highest education level, n (%)**								<.001
	Lower middle school degree or lower	6921 (75)	63 (56.3)	1671 (77)	5081 (75.7)	106 (46.5)		
	Upper middle school degree, technical or vocational degree	1807 (19.6)	36 (32.1)	374 (17.2)	1307 (19.5)	90 (39.5)		
	University or college degree or higher	477 (5.2)	12 (10.7)	121 (5.6)	312 (4.6)	32 (14)		
	Missing	22 (0.2)	1 (0.9)	5 (0.2)	16 (0.2)	0 (0)		

^a^Regions: The northern region comprises Beijing, Liaoning, and Heilongjiang. The eastern region comprises Shanghai, Jiangsu, and Shandong. The central region comprises Henan, Hubei, and Hunan. The western region comprises Chongqing and Guizhou, and the southern region comprises Guangxi.

^b^Regular intake defined as consumed specific food or drink more than 3 times per week.

### Association Between NCDs and Adhering to the Recommendations of 24HGs

In the overweight and obese participant group, after adjusting for sociodemographic and lifestyle behavioral factors, adhering to 24HGs was associated with a lower risk of NCDs. Compared with participants not adhering to any recommendations of 24HGs, those adhering to 1 (RR 0.37, 95% CI 0.19-0.74; *P*=.004), 2 (RR 0.37, 95% CI 0.19-0.72; *P*=.003), and all (RR 0.32, 95% CI 0.14-0.73; *P*=.006) components had a significantly lower risk of NCDs (Table 2).

**Table 2:** Association between the number of 24-hour movement guidelines met at baseline and the onset of chronic diseases in the overweight/obese group.

Adherence	Participants, n	Events, n	Model 1^a^			Model 2^b^			Model 3^c^	
			Risk ratio (95% CI)	*P* value	Risk ratio (95% CI)		*P* value	Risk ratio (95% CI)		*P* value
None	45	17	Reference	Reference	Reference		Reference	Reference		Reference
Meet 1 guideline	861	219	0.61 (0.44-0.94)	.01	0.37 (0.19-0.73)		.003	0.37 (0.19-0.74)		.004
Meet 2 guidelines	2757	659	0.61 (0.45-0.94)	.009	0.36 (0.19-0.70)		.002	0.37 (0.19-0.72)		.003
Meet all guidelines	105	22	0.48 (0.29-0.81)	.005	0.32 (0.14-0.73)		.006	0.32 (0.14-0.73)		.006

^a^Age*age and sex were adjusted; per score increase = 0.92 (95% CI 0.83-1.03); *P*=.14.

^b^The type of household register, province, survey year, smoking status, alcohol status, frequency of drinking sugar-sweetened beverage/tea/coffee, family net income, and light physical activity were additionally adjusted; per score increase = 0.88 (95% CI 0.75-1.03); *P*=.11.

^c^The whole movement time was additionally adjusted; per score increase = 0.89 (95% CI 0.76-1.04); *P*=.13.

In the normal weight or underweight group, compared with participants not adhering to any of the activity guidelines, those adhering to 1 (RR 0.49, 95% CI 0.27-0.96; *P*=.03) and overall components of the 24HGs (RR 0.40, 95% CI 0.17-0.94; *P*=.03) were associated with a lower risk of NCDs in the fully adjusted model. However, the association was not significant in the group adhering to 2 recommendations (Table 3).

**Table 3:** Association between the number of 24-hour movement guidelines met at baseline and the onset of chronic diseases in the normal/underweight group.

Adherence	Participants, n	Events, n	Model 1^a^		Model 2^b^			Model 3^c^	
			Risk ratio (95% CI)	*P* value	Risk ratio (95% CI)	*P* value	Risk ratio (95% CI)		*P* value
None	67	15	Reference	Reference	Reference	Reference	Reference		Reference
Meet 1 guideline	1310	230	0.55 (0.31-1.05)	.06	0.54 (0.30-1.04)	.053	0.49 (0.27-0.96)		.03
Meet 2 guidelines	3959	614	0.65 (0.37-1.23)	.16	0.63 (0.35-1.20)	.14	0.56 (0.30-1.09)		.07
Meet all guidelines	123	13	0.39 (0.17-0.89)	.03	0.43 (0.18-1.01)	.052	0.40 (0.17-0.94)		.03

^a^Age*age and sex were adjusted; per score increase = 1.03 (95% CI 0.89-1.20); *P*=.68.

^b^The type of household register, province, survey year, smoking status, alcohol status, frequency of drinking sugar-sweetened beverage/tea/coffee, family net income, and light physical activity were additionally adjusted; per score increase = 1.03 (95% CI 0.89-1.20); *P*=.66.

^c^The whole movement time was additionally adjusted; per score increase = 1.01 (95% CI 0.87-1.18); *P*=.90.

**Figure 1:**
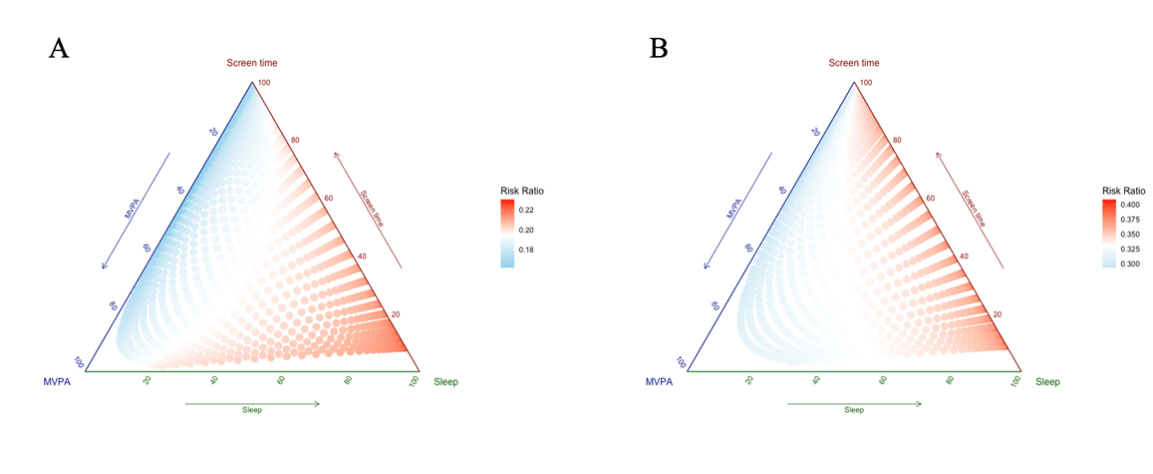
Heatmap ternary diagrams of expected risk ratios based on model 3 against different percentage time allocation of the movement behavior time-use composition in the (A) normal/underweight group and (B) overweight/obese group. MVPA: moderate to vigorous physical activity.

Additionally, we performed a compositional analysis with proportions of ratios of the actual time spent on each kind of activity and recommended time (the constituent ratios of 3 movements for each participant) to compare the results more comprehensively. Figure 1 shows the proportions of sleep, MVPA, and screen time, which are displayed using dotted blue and red lines, surrounded by 3 vertices in the heatmap. We observed a positive association between the proportion of screen time and risk of NCDs and a negative association between the proportions of MVPA the risk of NCDs.

### Stratified Analyses and Sensitivity Analyses

**Figure 2:**
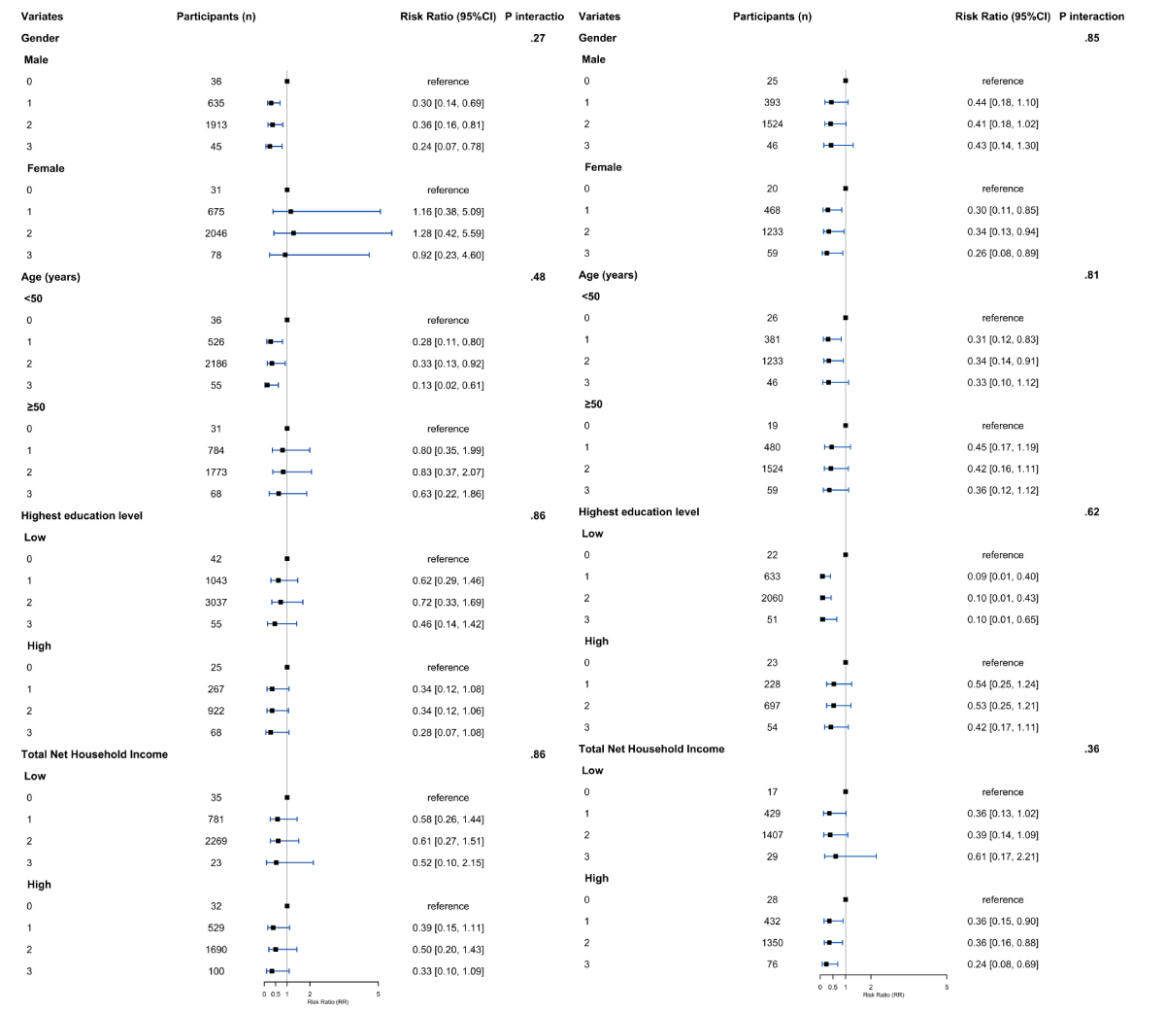
Subgroup analysis of the association between the number of 24-hour movement guidelines met at baseline and the onset of chronic diseases in the (A) normal/underweight group and (B) overweight/obese group.

For the specific combination of 24HGs, after fully adjusting for all covariates, we also observed an inverse association between adhering to 24HGs and risk for NCDs across specific combinations of the guidelines in the overweight and obese group. Compared with participants not adhering to any of the activity guidelines, those adhering to meeting screen time guideline (RR 0.34, 95% CI 0.18-0.69; *P*=.002), meeting screen time + MVPA guideline (RR 0.19, 95% CI 0.06-0.54; *P*=.002), meeting screen time + sleep guideline (RR 0.37, 95% CI 0.19-0.72; *P*=.003), and meeting all (RR 0.32, 95% CI 0.14-0.72; *P*=.006) had a significantly lower risk of NCDs (Table S1 of Multimedia Appendix 1). Moreover, in the subgroup analysis, we observed a similar inverse association between adhering to 24HGs and risk for overweight and obesity across age, sex, highest education level, and family net income subgroups in the fully adjusted model. We did not observe a significant interaction effect between these groups and adherence to 24HGs (Figure 2, Table S2 and Table S3 of Multimedia Appendix 1).

## Discussion

### Principal Findings

In this prospective cohort study, we found that compliance with 24HGs had a protective effect against major chronic diseases such as high blood pressure, stroke, diabetes, cancer, and acute myocardial infarction. These associations were independent of the potential confounding effects of age, sex, and socioeconomic status. Compared with individuals who met none of the recommendations, overweight or obese individuals who met 1, 2, or 3 recommendations had a 63%, 63%, and 68% lower risk of being diagnosed with NCDs, respectively. Restricted leisure screen time was observed to have a strong protective effect on chronic disease prevention. Among normal weight or underweight individuals, adhering to 1 or all components of 24HGs were linked with 51% or 60% lower risk of NCDs, respectively.

Although previous studies have performed several investigations regarding association between lifestyle behavior and life expectancy without chronic diseases [21,22], only few studies have assessed the compliance to 24HGs. Findings from a prospective multicohort study of 116,043 European adults aged 40-75 years showed that individuals who followed all the healthy lifestyle behaviors gained about 9 additional disease-free years of life compared to those who did not adopt any healthy behavior [23]. Nevertheless, the Nurses' Health Study and Health Professionals Follow-Up Study did not observe significant improvements in life expectancy upon adherence to more risk-reducing lifestyle behaviors [2]. This inconsistency in past evidence may be due to the inconclusive combination of healthy lifestyle behavior assessments [24,25]. In our study, 24-hour daily movements were considered an essential part of a healthy lifestyle, and the rationale for recommending 24HGs is already supported by evidence from past randomized controlled trials [26]. Furthermore, independent associations among adequate physical activity [4], limited leisure screen time, sleep duration [6,27], and NCD prevention have also been observed in previous observational and experimental investigations. However, previous studies have not been able to comprehensively assess the association between daily movements and NCDs, and only very few studies have evaluated the association between daily movements and the onset of major NCDs [28]. Our prospective investigation is thus able to expand upon these previous findings [23-28], clarifying that the association between adhering to overall recommendations of 24HGs and reduced risk of NCDs is observed in normal, overweight, and obese populations.

Daily movements and NCDs are plausibly linked biologically [29-32]. Adequate physical activity and appropriate sleep duration appear to interact with systemic bodily mechanisms relevant to inflammation, hemostasis, extracellular matrix mechanics, epigenetics, transcription, translation, circadian rhythms, exercise-induced muscle glucose uptake, microbiome, and lifestyle factors [29]. These interactions may underlie several NCDs, including cancer [29], diabetes [30], and stroke [31]. High levels of physical activity seem to mitigate the increased mortality risks associated with prolonged sedentary behavior [32]. Regular physical activity can also stimulate the immune system response and surveillance to help prevent cancer development [29]. Furthermore, limited sitting time has been associated with a lower risk of metabolic syndrome, insulin resistance, and improved C-reactive protein levels [29]. Specifically, promoting physical activity and reducing sedentary behavior during childhood and early adulthood may affect *BRCA1* mRNA expression in a way that could counteract the harmful effects of an inherited mutated *BRCA1* allele [29].

We observed a slight difference in RRs between the group with excess body weight and the group with normal/underweight. Overweight and obese individuals who met 1 or 2 or any type of activity guidelines were at a significantly lower risk of NCDs. However, this protective effect was not observed in individuals in the normal and underweight groups. Additionally, it is worth noting that point estimates among overweight and obese groups were significantly lower than those in the normal and underweight groups. Although the analysis of the interaction effect itself between BMI category and compliance with 24HGs was not significant (*P*=.55) in our analysis, we cannot prove that there is no potential interaction between BMI categories and daily movement. Indeed, significant interactions between lifestyle behaviors and BMI categories have been reported in the Nurses' Health Study and the Health Professionals Follow-up Study cohort studies [3]. The significantly lower risk of mortality and increase in health benefits of having any healthy lifestyle behavior has been observed particularly among individuals with BMI>24 [3].

Despite following the same recommendations of 24HGs, the normal/underweight and overweight/obese groups experienced different health benefits, as indicated by the point estimates. This divergence may be attributable to the particular vulnerabilities of the overweight/obese group, which are supported by well-established biological mechanisms. In comparison to the general population, obese individuals are at an increased risk. They are more susceptible to environmental risk factors for health, have a higher prevalence of mental health disorders [28], experience more substantial declines in quality of life due to chronic diseases [10], and face greater risks of COVID-19 hospitalization [33]. Due to the vulnerability of the obese population, even minor changes could lead to more significant and noticeable health improvements. The association between obesity and the onset of NCDs has potential biological mechanisms. A recent review [34] reported that groups with adiposity had long-term higher leptin or lower adiponectin concentrations, which might cause dysregulation of the immune system response that leads to abnormally higher concentrations of proinflammatory cytokines. Obesity is usually a situation of chronic low-grade systemic inflammation that leads to numerical and functional alterations of lymphocytes [35]. In the basal state, B-cell and T-cell responses are impaired in individuals with excess body weight, and these alterations may upsurge vulnerability to viral infection [35]. In addition, because several inflammatory components inhabit the tumor microenvironment and foster the phenotype of cancer, adipose inflammation is also considered to be a mediator of cancer development. In addition, the higher level of concentrations of adipocytokines are associated with fluctuations in the levels of angiotensinogen, plasminogen activator inhibitor-1, interleukin-6, and resistin, which might progress the onset of insulin resistance and atherosclerotic lesions [35,36]. Compared with general population, individuals with adipose inflammation and metabolic dysfunction exhibit higher cancer and cardiovascular disease risk [35,36]. For individuals who are overweight or obese, setting achievable goals and performing daily movements to intentionally lose weight not only reduce the levels of chronic disease-related factors but also reduce the risks of comorbidity incidence and disease progression. Adhering to physical activity recommendations may induce the beneficial effects of decrease in the levels of inflammatory biomarkers and improved endothelial function in obese children and adolescents [36].

Based on the analysis results of 59,005 adults in an English and Scottish Health Survey, vigorous-intensity leisure-time physical activity showed a significant effect on decreasing the cardiovascular disease mortality risk in the obese group [37]. Compared with active females with excess body weight, inactive females were exposed to 20%-41% higher risk of the onset of type 2 diabetes, while inactive males were exposed to 36%-38% higher risk than active overweight and obese males [38]. In addition, we found that meeting MVPA and leisure screen use recommendations would decline 81% risk of chronic diseases. Besides the salutary benefits conferred by physical activity, our analysis revealed the concurrent reduction in the risk of NCD onset when leisure screen time is curtailed, particularly when combined with adequate sleep duration. The nonsignificant values observed in the results for the association between MVPA and NCD risk may be attributable to the smaller sample size rather than a lack of association between MVPA and the prevention of NCDs. A smaller sample size can diminish the statistical power of the study, potentially leading to a type II error, where a true effect is present but not detected. Thus, the observed significance value should not be interpreted as definitive evidence of no relationship between MVPA and NCD prevention. Further research with a larger sample size is warranted to accurately determine the extent of this association. Future research is needed to investigate the mechanisms that link adhering to 24HGs with NCD risk as well as to explore effective daily movement interventions needed for substantial NCD risk reduction.

### Strengths and Limitations

One strength of our study was that our analysis was based on a large nationwide sample size of adults with long-term follow-up data, which provided high statistical power and the ability to infer temporality. Another important strength was that the data collection of CHNS was conducted under robust quality control management and performed by qualified staff.

There are, however, several limitations to our study. First, the data on lifestyle behaviors were collected via self-reported questionnaires, which might be subject to misclassification or self-report biases. Nonetheless, the results of our study were largely consistent with past research [2,23-28], supporting the validity of our findings. Second, there are likely various potential unmeasured factors related to daily activities or NCD status that could not be adjusted for in the analyses. Third, in the CHNS, sleep duration was collected through self-reported questionnaires. Therefore, we were unable to assess participants’ sleep quality, as sleep quality is typically measured by electroencephalography monitoring, which would not have been feasible in a population-based survey, given its complexity and cost. Thus, in our study, for meeting the guidelines, we were primarily concerned with the duration rather than quality. Fourth, the outcome data on NCDs were collected through self-reported questionnaires. Although participants reported health conditions based on their recollection of hospital diagnoses and medical records, the staff did not have access to the exact diagnosis dates. As a result, the ascertainment of NCDs relied on self-reported data, which may have introduced recall biases. Finally, as we enrolled only Chinese adults in our cohort study, our estimates may not be generalizable to other population subgroups with different social structures and characteristics.

### Conclusion

Based on the CHNS data from the 2004 to 2015 waves, our study shows that actively following 24HGs was associated with lower risks of NCDs, especially among overweight or obese individuals. Furthermore, overweight and obese individuals who met at least 1 component of 24HGs were at a significantly lower risk of NCDs. However, this protective effect was not observed in normal or underweight groups. Individuals with excess body weight who tend to be prone to greater health risks may gain larger health benefits by adhering to these recommendations compared to the general population.

## Appendix

Multimedia Appendix 1 Supplementary data.

## Abbreviations

24HGs: Canadian 24-Hour Movement Guidelines
CHNS: China Health and Nutrition Survey
MVPA: moderate to vigorous physical activity
NCD: noncommunicable disease
RR: risk ratio

## Data Availability

The data sets generated and analyzed during this study were sourced from the China Health and Nutrition Survey. Researchers can access the data on the official China Health and Nutrition Survey website [39] after submitting an application. The data sets used in this study are also available from the corresponding author upon reasonable request.
