# Supplementary material for: Association Between Adherence to 24-Hour Movement Guidelines and Noncommunicable Disease Risk in Chinese Adults: Prospective Cohort Study
Source: JMIR Public Health Surveill. 2024 Mar 27;10:e47517. doi: 10.2196/47517 (PMC11007614; doi:10.2196/47517)
Supplement: Multimedia Appendix 1 [file publichealth_v10i1e47517_app1.docx]

**Table S1. Sensitivity analysis for the association between adhering to specific combination of 24-hour movement guidelines and the onset of chronic diseases among overweight/obesity group**

| **Adhering to guidelines** | **Participants** | **Events** | Model 1^a^ | | Model 2^b^ | | Model 3^c^ | |
| --- | --- | --- | --- | --- | --- | --- | --- | --- |
|  |  |  | **RR (95%CI)** | ***P* value** | **RR (95%CI)** | ***P* value** | **RR (95%CI)** | ***P* value** |
| **None** | 45 | 17 | Reference | | Reference | | Reference | |
| **Meet MVPA** | 6 | 3 | 1.07 [0.33, 2.04] | .87 | 0.88 [0.14, 5.62] | .89 | 0.88 [0.14, 5.59] | .89 |
| **Meet Screentime** | 697 | 180 | 0.60 [0.43, 0.92] | .008 | 0.34 [0.18, 0.68] | .002 | 0.34 [0.18, 0.69] | .002 |
| **Meet Sleep** | 158 | 36 | 0.66 [0.42, 1.07] | .07 | 0.50 [0.24, 1.07] | .07 | 0.50 [0.23, 1.06] | .07 |
| **Meet mvpa+screentime** | 40 | 7 | 0.38 [0.16, 0.76] | .01 | 0.19 [0.06, 0.54] | .002 | 0.19 [0.06, 0.54] | .002 |
| **Meet MVPA+Sleep** | 14 | 2 | 0.33 [0.06, 0.94] | .10 | 0.24 [0.03, 1.06] | .09 | 0.24 [0.03, 1.06] | .09 |
| **Meet Screentime+Sleep** | 2703 | 650 | 0.62 [0.45, 0.94] | .01 | 0.36 [0.19, 0.71] | .002 | 0.37 [0.19, 0.72] | .003 |
| **Meet All** | 105 | 22 | 0.48 [0.29, 0.81] | .005 | 0.32 [0.14, 0.72] | .006 | 0.32 [0.14, 0.72] | .006 |

a: Age, age*age and sex were adjusted

b: The type of household register, province, survey year, smoking status, alcohol status, frequency of drinking sugar beverage, tea or coffee, family net income, and light physical activity were additionally adjusted.

c: The whole movement time was additionally adjusted

**Table S2. Sensitivity analysis for the association between number of 24-hour movement guidelines met at baseline and the onset of chronic diseases among normal weight group**

| **Adhere to guidelines** | **No. of Participants** | **No. of Events** | **Model 1^a^** | | **Model 2^b^** | | **Model 3^c^** | |
| --- | --- | --- | --- | --- | --- | --- | --- | --- |
|  |  |  | **RR (95%CI)** | ***P* value** | **RR (95%CI)** | ***P* value** | **RR (95%CI)** | ***P* value** |
| **None** | 62 | 15 | Reference | | Reference | | Reference | |
| **Meet one** | 1196 | 213 | 0.50 [0.28, 0.97] | .03 | 0.49 [0.27, 0.96] | .03 | 0.45 [0.24, 0.89] | .02 |
| **Meet Two** | 3695 | 581 | 0.60 [0.33, 1.13] | .10 | 0.58 [0.32, 1.11] | .08 | 0.51 [0.27, 1.01] | .04 |
| **Meet All** | 119 | 13 | 0.36 [0.16, 0.84] | .02 | 0.40 [0.17, 0.93] | .03 | 0.37 [0.15, 0.87] | .02 |
| **Per score increase** | |  | 1.02 [0.88, 1.19] | | 1.02 [0.88, 1.20] | | 1.00 [0.85, 1.18] | |
| ***P* for trend** | |  | 0.804 | | 0.784 | | 0.987 | |

a: Age, age*age and sex were adjusted

b: The type of household register, province, survey year, smoking status, alcohol status, frequency of drinking sugar beverage, tea or coffee, family net income, and light physical activity were additionally adjusted.

c: The whole movement time was additionally adjusted

**Table S3. Subgroup analysis for the association between number of 24-hour movement guidelines met at baseline and the onset of chronic diseases among normal/underweight group**

| **Variable** | **Category of adhering to 24-hour movement guidelines** | | | | |
| --- | --- | --- | --- | --- | --- |
|  | **None (n=67)** | **Meet one standard (n=1310)** | **Meet two standards (n=3959)** | **All meet (n=123)** | ***P* value for interaction** |
| **Sex** |  |  |  |  | .27 |
| **Male** | 1 [Reference] | 0.30 [0.14, 0.69] | 0.36 [0.16, 0.81] | 0.24 [0.07, 0.78] |  |
| **Female** | 1 [Reference] | 1.16 [0.38, 5.09] | 1.28 [0.42, 5.59] | 0.92 [0.23, 4.60] |  |
| **Age** |  |  |  |  | .48 |
| **<50** | 1 [Reference] | 0.28 [0.11, 0.80] | 0.33 [0.13, 0.92] | 0.13 [0.02, 0.61] |  |
| **>=50** | 1 [Reference] | 0.80 [0.35, 1.99] | 0.83 [0.37, 2.07] | 0.63 [0.22, 1.86] |  |
| **highest education level** | |  |  |  | .86 |
| **Low** | 1 [Reference] | 0.62 [0.29, 1.46] | 0.72 [0.33, 1.69] | 0.46 [0.14, 1.42] |  |
| **High** | 1 [Reference] | 0.34 [0.12, 1.08] | 0.34 [0.12, 1.06] | 0.28 [0.07, 1.08] |  |
| **Family net income** | |  |  |  | .86 |
| **Low** | 1 [Reference] | 0.58 [0.26, 1.44] | 0.61 [0.27, 1.51] | 0.52 [0.10, 2.15] |  |
| **High** | 1 [Reference] | 0.39 [0.15, 1.11] | 0.50 [0.20, 1.43] | 0.33 [0.10, 1.09] |  |

**Table S4. Subgroup analysis for the association between number of 24-hour movement guidelines met at baseline and the onset of chronic diseases among overweight/obesity group**

| **Variable** | **Category of adhering to 24-hour movement guidelines** | | | | |
| --- | --- | --- | --- | --- | --- |
|  | **None (n=45)** | **Meet one standard (n=861)** | **Meet two standards (n=2757)** | **All meet (n=105)** | ***P* value for interaction** |
| **Sex** |  |  |  |  | .85 |
| **Male** | 1 [Reference] | 0.44 [0.18, 1.10] | 0.41 [0.18, 1.02] | 0.43 [0.14, 1.30] |  |
| **Female** | 1 [Reference] | 0.30 [0.11, 0.85] | 0.34 [0.13, 0.94] | 0.26 [0.08, 0.89] |  |
| **Age** |  |  |  |  | .81 |
| **<50** | 1 [Reference] | 0.31 [0.12, 0.83] | 0.34 [0.14, 0.91] | 0.33 [0.10, 1.12] |  |
| **>=50** | 1 [Reference] | 0.45 [0.17, 1.19] | 0.42 [0.16, 1.11] | 0.36 [0.12, 1.12] |  |
| **highest education level** | |  |  |  | .62 |
| **Low** | 1 [Reference] | 0.09 [0.01, 0.40] | 0.10 [0.01, 0.43] | 0.10 [0.01, 0.65] |  |
| **High** | 1 [Reference] | 0.54 [0.25, 1.24] | 0.53 [0.25, 1.21] | 0.42 [0.17, 1.11] |  |
| **Family net income** | |  |  |  | .36 |
| **Low** | 1 [Reference] | 0.36 [0.13, 1.02] | 0.39 [0.14, 1.09] | 0.61 [0.17, 2.21] |  |
| **High** | 1 [Reference] | 0.36 [0.15, 0.90] | 0.36 [0.16, 0.88] | 0.24 [0.08, 0.69] |  |
